# Screening Depressive Disorders With Tree-Drawing Test

**DOI:** 10.3389/fpsyg.2020.01446

**Published:** 2020-06-25

**Authors:** Simeng Gu, Yige Liu, Fei Liang, Rou Feng, Yawen Li, Guorui Liu, Mengdan Gao, Wei Liu, Fushun Wang, Jason H. Huang

**Affiliations:** ^1^Department of Psychology, Medical School, Jiangsu University, Zhenjiang, China; ^2^Department of Business Studies and Economics, University of Gävle, Gävle, Sweden; ^3^Department of Psychology, Nanjing University of Chinese Medicine, Nanjing, China; ^4^Institute of Brain and Psychological Sciences, Sichuan Normal University, Chengdu, China; ^5^Suzhou Guangji Hospital, Suzhou, China; ^6^Department of Neurosurgery, Baylor Scott & White Medical Center – Temple, Temple, TX, United States; ^7^Department of Surgery, Texas A&M University College of Medicine, Temple, TX, United States

**Keywords:** tree-drawing test, affective disorders, depression, major depressive disorders, quantitative study, emotion

## Abstract

**Objective:** Diagnosis of psychiatric disease is still a major issue. Two key reasons are- there are variations in the opinions of the medical doctors and the presentation of a disease among the patients. Here we introduce a kind of mental projective test, tree-drawing test, trying to extract and analyze objective indexes in tree-drawing test in patients with depression.

**Methods:** The tree-drawing test was administered to 43 patients with major depressive disorders, 48 sub-threshold subjects, and 59 healthy subjects. Features of the drawing trees were analyzed using a kind of computer image recognition and data acquisition software. Quantitative indexes collected from pictures drawn by patients with major depression, patients with sub-threshold depression, and control subjects were compared using the ANOVA test.

**Results:** Five quantitative features (canopy area, canopy height, canopy width, trunk width, and total area of trees) were found to be statistically significant among the groups, while seven other features (trunk area, trunk height, root width, root height, root area, ratio of crown to trunk height, and ratio of crown to trunk area) showed no statistical significance. Further analysis with LSD-t test revealed that six quantitative indexes were significantly related to the depression symptoms, and six others were not statistically significant. Eleven quantitative indexes were not statistically significant when the depressive symptoms were compared with the subthreshold depression group, and the only index with statistical significance was canopy width.

**Conclusion:** Five quantitative indexes in the drawing tree are statistically significant in the depression patients were compared with those of the control subjects. Quantitative indexes of the tree-drawing test are of great value in assisting with the diagnosis of psychiatric disorders.

## Introduction

Major depressive disorder is affecting more than 10% of populations worldwide, and the World Health Organization anticipated that it will be the first health problem in 2030 (Gu et al., 2018). However, diagnosis of depression (like many other psychiatric diseases) is still a major issue, and the two key reasons are: there are variations in the opinions of the medical doctors and the presentation of a disease among the subjects (Mistra et al., 2012). Current diagnoses, which are typically viewed as the “golden standard” (such as low mood, no interest, thought retardation, no motivation, thinking of suicide), depend on interview and self-rated questionnaires (Davison et al., 2009). Even though the criteria of diagnostic manuals, like DSM and ICD, is getting more and more detailed, some reports suggested that the diagnosis of depression is substantially underdiagnosed in primary care (Schwartz et al., 2019), or the rate of diagnosis of depression in non-depressed patients was estimated to be as high as 26.5% (Aragones et al., 2011). Some papers even suggested that clinician-based standardized diagnoses are not feasible, even not as good as self-reported questionnaire (Fisher et al., 2015). In addition, BD is often misdiagnosed as Major Depression Disorder (MDD), with approximately 40% of BD patients being initially diagnosed as MDD (Correia et al., 2009). These proposals suggest a need for definitive and truly objective physical or chemical index, or a judicious selection of MDD case-finding instruments depending on the study population and target periods of assessment (Owora et al., 2016).

So there are many seeks for new ways to diagnose depression, such as EEG based depression recognition (Li et al., 2019), or voice acoustics (Hashim et al., 2017). EEG is might be a good biomarker for depression, for example, a recent EEG study suggests that P1 amplitude to sad face showed potential as a state marker of depression (Ruohonen et al., 2020). Voice acoustic features extracted from reading speech demonstrated variable effectiveness in predicting clinical depression scores. Voice features were highly predictive of Hamilton depression. The methodology is feasible for diagnostic applications in diverse clinical settings as it can be implemented during a standard clinical interview in a normal closed room and without strict control on the recording environment.

Tree drawing is one kind of mental projective test too, which refers to the free expression of thoughts that can then be interpreted to reflect inside thoughts of the subject (Wang et al., 2014). Projective tests usually employ ambiguous stimuli, notably inkblots to evoke responses that may reveal facets of the subject’s personality by projection of the internal thoughts, for example, Rorschach inkblot test is a kind of test that includes 10 irregular but symmetrical inkblots, and asks the subjects to explain what they see. The subject’s responses are then analyzed in various ways, such as what was said, the time taken to respond, which aspect of the drawing was focused, etc. Other projective methods involve requiring the subjects to build wooden block structures, complete sentences, tree-drawings. The results are based on psychdynamic interpretation of the details of the drawing, such as size, shape, complexity of the tree. Projective test is a personality test designed to reveal hidden emotions and internal conflicts. The tree drawing test is easy to use and has less stress on the patients, and most importantly, the patients cannot easily hide their emotions, because they do not know which feature represents depression, unlike that in the questionnaire report.

Tree drawing test is a very useful tool in the differential diagnosis of mental health and the qualitative evaluation of treatment outcomes (Igimi et al., 2001; Morita et al., 2001). The tree-drawing test was developed in 1952, and quickly attracted the attention of researchers and has been widely used by clinicians (Hu and Chen, 2012). There were extensive researches of tree drawing test in the psychological field which demonstrated that tree drawing has a good ability to distinguish between pathological condition and normal condition (Kan and Guangxing, 2008; Kaneda et al., 2010). According to domestic and foreign literature reports, the tree-drawing test is reliable and valid (Chen and Xu, 2008a) and some scholars have used the tree-drawing tests in the diagnosis of neurosis, depression, and found some indexes are reliable markers for these mental disorders (Chen et al., 2011; Cai et al., 2012). The tree-drawing tests have been used in emotional test, psychological screening, and post-disaster relief (Chen and Xu, 2008b), and have been proved to be a useful tool for the diagnosis of emotional disorders (Inadomi et al., 2003).

Some studies have reported that certain common indexes of tree drawings are linked to emotional disorders. For example, some investigators reported that certain qualitative indicators of the tree-drawings differ in patients with mental health problems from that in healthy people, and they found eight drawing characteristics have a high level of diagnosis utility, and suggested that the tree drawing test is of some value for diagnosis of depression in adolescents (Inadomi et al., 2003). And it has been suggested that the values concerning the size of the tree, such as the height and width of the whole tree, height and width of the crown, and number of occupied areas (of the paper), were significantly lower in the depression (Murayama et al., 2016). However, these indicators are relatively primitive and existing studies have only addressed the correlation between the tree-drawing test and mental health problems. In this study, we used an impartial quantitative analysis to collect data from the tree-drawings. We used image scanning and computer image recognition technology to quantitatively collect the height and width of drawn trees, such that the size of the tree canopy, trunk, and roots were also determined from a scanned image. Therefore, we compared and analyzed the differences between the tree-drawing test indexes of patients with major depression in order to explore the in-depth putative diagnostic indexes of major depression, and also to evaluate the effectiveness in its clinical diagnostic application. This may help determine whether tree drawing tests can be introduced as an identifier of sub-threshold and depression in clinical psychology. In addition, it may be possible to use these quantitative indexes as an auxiliary diagnostic tool for the identification of various emotional disorders.

## Patients and Methods

### Patients

Major depressive patients were clinically diagnosed patients, with stable symptoms and features, and they were recruited from the Department of General Psychiatry of Zhenjiang Mental Health Center from February 2017 to November 2018. These patients were newly admitted to and resided at the Center for depression. The inclusion criteria were: (1) patient met the DSM-5 mental and behavioral disorder classification for diagnosis of depression (Fan, 1993); (2) age 18–60 years old, no gender limits; (3) Hamilton Depression Scale (HAMD) score, 24 items ≥20 points (He, 1999; Zhang and He, 2015); (4) patients were selected between days 3 and 7 post-admission, i.e., during the symptomatic period. Exclusion criteria: (1) pregnant, lactating, or menopausal women; (2) psychoactive substance abuse and other severe psychiatric disorders; (3) patients with severe unstable physical diseases, diagnosed diabetes, thyroid disease, or hypertension. In this study, there were 43 patients with major depressive disorders, 14 males and 29 females, aged 18–49 years, with an average age of 34.3 ± 7.8. Patients with depression were hospitalized for 4–6 weeks. Medications used include: escitalopram, sertraline, and venlafaxine.

**FIGURE 1:**
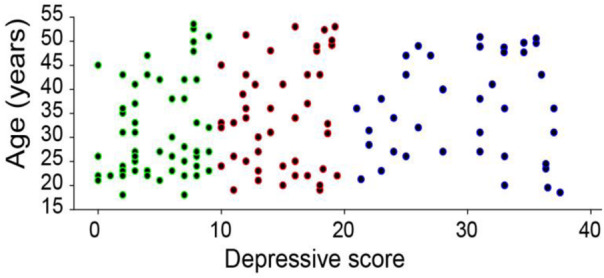
The distribution of the subjects and their scores in Hamilton Depression Scale. The control group scored less than 10, while the sub-threshold group scored higher than 10, and the MDD group scored higher than 20. The average age for control was 32.3 ± 8.9 (*n* = 59), sub-threshold group was 35.3 ± 8.6 (*n* = 48), and MDD group was 34.3 ± 7.8 (*n* = 43). And the mean score from Hamilton was 4.44 ± 2.6, 14.5 ± 3.5, 28.1 ± 5.4 for the three groups, respectively.

The sub-threshold depressive group and control group included subjects residing in the same region as the patients and were enrolled in this study at the same time. Enrollment criteria: (1) no obvious symptoms of psychiatric disorders (SCL-90, no positive factor), no previous history of mental illness; (2) match the gender of the experimental group; (3) did not receive any training in drawing. Exclusion criteria were the same as the patient group. The sub-group and the normal group were divided with the Hamilton score (Figure 1), and got 48 subjects in the sub-threshold group and 59 subjects for the control group. The sub-threshold group includes 23 males and 25 females, aged 18–55 years old (35.3 ± 8.6). The control group include were 26 males and 33 females, aged 18–50 years, with average age of 32.3 ± 8.9 years (Figure 1).

### Research Tools

#### Tree-Drawing Test

In order to make the experimental procedures standardized, we used the same procedure as reported before (Wang et al., 2014). Briefly, each participant was provided with A4 paper and a black or blue-black pen. Participants were instructed to draw a tree following these five rules: (1) The tree-drawing test is not a test of drawing technique and the drawing does not need to be aesthetically pleasant; (2) The tree does not need to appear life-like; (3) If you want to draw something that you are not capable of drawing, you can draw a circle and identify the intended object in writing with words; (4) Before you draw a tree, close your eyes and meditate for half a minute. Draw the tree that appears in the meditation. If there is no tree in the meditation, open your eyes and draw the tree that is most appealing to you; (5) After completing the drawing, write down your age, gender, and occupation at the bottom of the paper.

In addition, in the process of collecting the drawn trees, each drawing was inspected. If some features or indexes were not immediately identifiable, the drawing was discarded from the analysis. In all of the drawings collected, there were two drawings from control group and three drawing from the MDD group were discarded.

#### High-Definition Scanning

The Epson GT-1500 HD scanner was used to scan drawn trees and to save scanned images.

#### Data Collection

**FIGURE 2:**
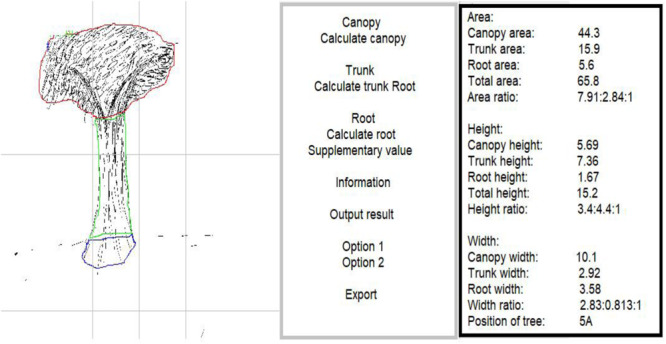
Example of scanned tree in major depression group.

**FIGURE 3:**
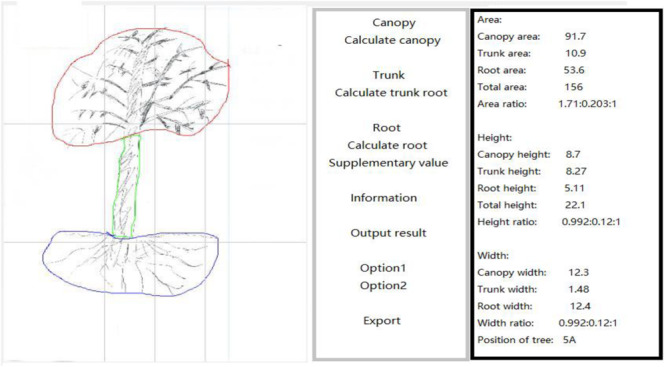
Example of scanned tree in control group.

R&D image data scan acquisition software (Figures 2, 3) was used to extract data. The project of developing software to analyze drawn trees was developed in 2015–2017, with technical support provided by the School of Computer Science of Jiangsu University. The software was licensed by the National Copyright Administration in July 2017 (Liu, 2017). The software automatically and accurately extracts tree length, width, height and area data, and calculates the proportion of each part.

### Statistical Analysis

All data were shown as mean ± standard deviation, and analyzed using SPSS 17.0 statistical software. We used one-way ANOVA and LSD-t test for analysis.

## Results

### Size of the Canopy, Trunk, Roots, and the Entire Tree

**FIGURE 4:**
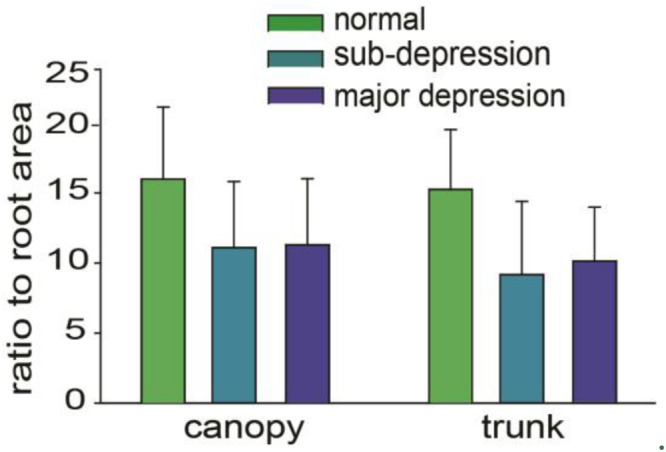
Comparisons among the ratios of canopy area, trunk area to root area.

After the data from drawn trees were collected, statistical software was used to perform one-way ANOVA test. We first analyzed the ratios of the canopy area and trunk area to the root area, as it was reported before that the ratio between canopy and trunk as well as roots was different in the depression group. However, even though the ratio of canopy to root is higher than that of the depression group and sub-depression group (Pintea et al., 2014), they are not significantly different among the groups (*p* > 0.05, one-way ANOVA, Figure 4). The differences in the size of canopy, trunk and total tree area among the three groups were statistically significant (*p* < 0.05, one-way ANOVA). There was no significant difference between the depression group and the sub-threshold group in canopy area, trunk area and total area of trees (*p* > 0.05, one-way ANOVA). However, there were significant differences between the major depressive group with the control group in canopy area, trunk area, and total area of drawn trees (*p* < 0.01, one-way ANOVA, Table 1).

**TABLE 1:** Size (cm^2^) of canopy, trunk, roots, and total tree in the groups.

Features	Canopy area	Trunk area	Root area	Total tree area
Depression	45.18 ± 42.34**	9.16 ± 9.64**	2.61 ± 7.59	56.76 ± 49.96**
Sub-threshold	55.17 ± 51.28*	16.80 ± 7.81*	4.39 ± 3.81	98.45 ± 23.43*
Normal	90.83 ± 61.96	19.33 ± 6.47	4.37 ± 12.46	114.52 ± 73.87
*F*	8.22	5.23	0.85	10.32
*P*	0.000	0.001	0.67	0.000

**p < 0.01, *p < 0.05, post-hoc results, compared with control.

### Height of the Canopy, Trunk, Roots, and the Whole Tree

**FIGURE 5:**
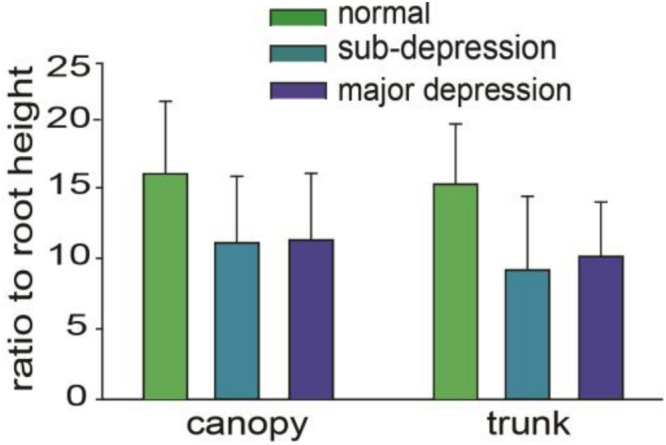
Percentage of canopy, trunk, and root height in the total height. The differences were not statistically significant (*p* > 0.05, one way ANOVA).

There were significant differences among the three groups in canopy height and in the height of whole trees (*p* < 0.05, one-way ANOVA, *n* = 48 = 59). The results of the analysis were post-tested. The difference in canopy height between the depression group and the sub-threshold group was statistically significant. The difference in canopy height between the depression group and the control group, as well as the difference between the sub-threshold group and the control group were statistically significant (*p* < 0.01). As for the total height of the tree, there were significant differences between the depression group and the sub-threshold group, as well as between the depression group and the control group. It was not significantly different between the sub-threshold group and the control group (*p* > 0.05, one-way ANOVA, Table 2). The ratios of canopy height, trunk height to root height were not significantly different either (Figure 5).

**TABLE 2:** Height (cm) of canopy, trunk, root, and whole tree in the groups.

Feature	Canopy height	Trunk height	Root height	Total tree height
Depression	6.22 ± 3.34**	5.01 ± 3.27	0.51 ± 0.99	11.74 ± 5.48**
Sub-threshold	7.56 ± 3.87*	6.66 ± 4.78	0.78 ± 0.74	13.46 ± 6.39*
Normal	8.72 ± 3.62	6.43 ± 3.02	0.47 ± 1.09	15.63 ± 5.41
*F*	6.16	1.95	0.844	4.754
*P*	0.003	0.21	0.237	0.007

*p < 0.05, **p < 0.01, post-hoc results, compared with control.

### Width of the Canopy, Trunk, and Root

**FIGURE 6:**
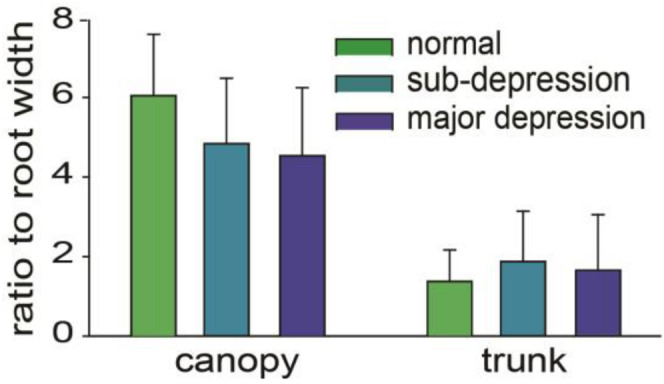
Ratio of canopy width and trunk width to root width, the differences were not statistically significant (*p*> 0.05, one way ANOVA).

There were statistically significant differences among the three groups in the width of the canopy and the width of the trunk (*p* < 0.01, one way ANOVA, *n* = 48–59). *Post-hoc* results of the analysis showed that the difference in width of the canopy was statistically significant between the three groups (*p* < 0.01). The depression group, the control group, and the sub-threshold group show a statistically significant difference in the width of the trunk as well (*p* < 0.01). It was not significantly different in the width of the trunk between the depression group and the sub-threshold group (Table 3). The ratios of canopy width, trunk width to the root width were not significantly different either (Figure 6).

**TABLE 3:** Canopy, trunk, and root width (cm) in the groups.

Quantitative index	Canopy width	Trunk width	Root width
Depression group	7.53 ± 3.84**	1.67 ± 1.39**	1.29 ± 2.93
Sub-threshold group	10.54 ± 4.89*	3.38 ± 1.79	1.67 ± 2.32
Control	12.10 ± 4.78	3.73 ± 2.28	2.20 ± 4.82
*F*	14.28	11.75	0.966
*P*	0.000	0.000	0.517

*p < 0.05, **p < 0.01, post-hoc results, compared with control.

### Logistic Regression Analysis of Symptoms of Depression and Sub-Threshold

Quantitative indexes were selected for Logistic stepwise regression analysis. The respondent variable is “Y,” *Y* = 1 is a patient with depression, *Y* = 2 is a sub-threshold patient, *Y* = 3 is a control subject; the independent variable is “X,” X1–X11 represent 11 quantitative indexes of drawn trees. The independent variable assignment is shown in Table 4.

**TABLE 4:** Variable assignment description (*n* = 145).

Variable	Quantitative index
X1	Canopy area
X2	Trunk area
X3	Root area
X4	Total area
X5	Canopy height
X6	Trunk height
X7	Root height
X8	Total height
X9	Canopy width
X10	Trunk width
X11	Root width

Logistic stepwise regression analysis revealed that there were seven quantitative indexes of the depression group in the regression equation, while there were four quantitative indexes of the sub-threshold group in the regression equation, *X*^2^ = 73.564, *P* = 0.000, and the Logistic regression equation was statistically significant. The results are shown in Table 5.

**TABLE 5:** Logistic regression analysis of the quantitative indexes of tree drawings in depression and sub-threshold.

Group	Characteristic	B	S.E.	Wald	df	Sig	Exp(B)
Depression group	X1	–3.18	1.23	6.650	1	0.010	0.041
	X2	–3.08	1.22	6.326	1	0.012	0.046
	X3	–3.17	1.23	6.636	1	0.010	0.010
	X4	+ 3.20	1.23	6.731	1	0.009	0.009
	X7	+ 3.02	1.42	4.645	1	0.031	20.517
	X9	–0.29	0.13	4.912	1	0.027	0.742
	X10	–0.97	0.32	9.311	1	0.002	0.376
	Constant	+ 2.87	1.09	6.908	1	0.009	
Sub-threshold group	X4	+ 1.83	0.93	3.836	1	0.050	0.162
	X9	–0.35	0.32	5.86	1	0.054	084
	X10	–0.71	0.35	9.02	1	0.004	0.500
	X11	–0.85	0.47	4.42	1	0.026	0.398
	Constant	+ 3.25	1.29	4.76	1	0.018	

### Establish Regression Equations Based on Logistic Regression Analysis

From the regression coefficients in Table 4, the regression equations of the quantitative indexes for symptoms of depression and symptoms of sub-threshold can be obtained, and the Nagelkerke R2 coefficient test can be performed to explain the regression equation. The regression equation of drawn tree quantitative indexes for depressive symptoms is: ln($\frac{Y=1}{Y=3}$) = 2.877 − 3.186 X1 − 3.085 X2 − 3.173 X3 + 3.2 X4 + 3.021 X7 − 0.298 X9 − 0.978 X10; The regression equation of quantitative indexes on sub-threshold symptoms is: ln($\frac{Y=2}{Y=3}$) = 2.395 + 1.839 X4 − 0.252 X9 − 0.694 X10 − 0.921 X11. The Nagelkerke R2 value is 0.395, indicating that the quantitative indexes are of an acceptable level.

## Discussion

Diagnosis of depression is still a major issue, and it is important to find more biomarkers for the diagnosis (Liu, 2017). The tree-drawing test is already in widespread use amongst psychiatric occupational therapists in Taiwan, but studies about objective standards are somewhat limited (Pintea et al., 2014), and many characteristics have been suggested to be related to many mental diseases (Gu et al., 2016). Results from this study show that there are statistical differences in the selected quantitative indexes of canopy area, canopy height, canopy width, trunk width, and total area of trees among the patients in the major depressive group, sub-threshold group and the control group. In addition they are co-related with the severity of the depression. However, there were no significant differences in trunk area, trunk height, root width, root height, root area, height of canopy to trunk, and area of canopy to trunk. These findings indicate that selected quantitative indexes from the tree-drawing test can be used as an auxiliary diagnostic tool to screen depressive disorders. This is consistent with previous reports that canopy mainly reflects the state of the patient’s emotional state of the person, and the root of the tree mainly reflects the subject’s unconscious instincts (Wang et al., 2014). Thus the tree drawing test can be used to diagnose depression through the size of the canopy, and also the ratio between crown and trunk (Li et al., 2011).

### Analysis of Canopy of the Trees

The canopy is the part of the tree that is used to connect with the external environment and to exchange with the outside world. Therefore, the canopy was suggested to reflect mainly the conscious emotional state of the subjects (Kaneda et al., 2010). It is suggested that the canopy expresses the subjects’ understanding and structure of his or her intimate relationship with family, relatives, and others. In addition, the canopy also describes the spiritual and emotional intellectual development of the subject, scope of interest, goals and aspirations, and their overall satisfaction (Kaneda et al., 2010). The height, width and area of the canopy in the depression group were much smaller than those of the control group. This is consistent with symptoms of depression, helplessness, feelings of uselessness, lack of interest, loss of interest, and pessimism. In this study, the total area of trees in the depression group was significantly smaller than that in the control group, and the difference was statistically significant. The results of this study also show that the height, width and size of the canopy in the depression group were smaller than those in the control group, and the difference was statistically significant. It has been pointed out that the size of trees directly reflects the current overall state of the individual. Tall trees indicate individuals with better development, high mental energy levels, sufficient self-confidence, etc. When trees are short, subjects are often found to have depression (Yan and Chen, 2011; Hu and Chen, 2012). In all, the results of the tree-drawing test in patients with depression reflect their personality traits, including depressed motivation, low self-esteem, lacking of vitality, lacking of self-control, dependence, withdrawal, disengagement, and self-destruction (Ji, 2011).

### Analysis of Canopy of the Trees Trunk

**FIGURE 7:**
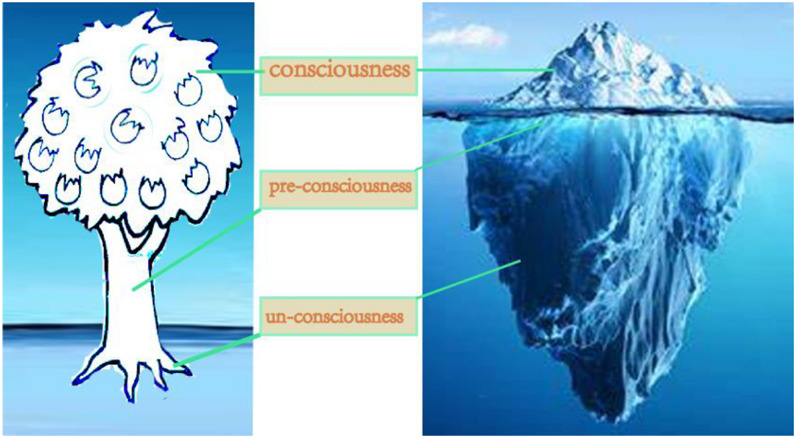
Hypothesis about the representation of a tree-drawing with the consciousness.

The area, height and width of the trunk of the depression group were also smaller than those of the control group, and the differences in the three indexes were statistically significant. This is possibly due to the proposal that trunk is correlated with the inner emotional function of the person and represents the unconscious emotion state of the person (see Figure 7). The width of the trunk can be seen as an indicator of emotional depth, while the length represents the degree of domination of emotional mood (Ji, 2011). The core symptoms of depression patients include moodiness or depression, lack of interest, and loss of interest (Xie and Ye, 1994). The small trunk area of patients with depression reflects their negative mood and pessimism; the short trunk reflects their poor emotional control; the narrow width reflects their emotional vulnerability (Matt, 2015). An appropriate trunk area, height, and width might indicate a stable mood.

The results of our study also show that there are significant differences between the tree area and the trunk width of trees drawn by depressive patients and control subjects. Matt Rowley believes that the trunk is the most important part of a tree, connecting the roots and crowns, and feeding the branches and leaves, and that it is the foundation of a tree (Xie and Ye, 1994). In the tree-drawing test, the trunk is possibly the symbol of the inner self and symbol of emotion. Although disordered thinking is the most prominent manifestation of emotional disorder, it is also an important component and mainly manifested as emotional apathy: a cold and dull emotional response to surroundings, or emotional inversion, emotional dissonance, etc. Most MDD patients do not perceive their loss of emotional expression. Compared with that of the control group, the small trunk of depressive patients reflects the characteristics of affective disorders such as apathy, active requirements, reduced hobbies, and other emotional impediments. A wide trunk indicates long-term mood stability. The width of the trunk in patients with sub-threshold is smaller than that of the control group, indicating that their emotional response shows superficial and uncoordinated characteristics. Other studies have found that the ratio of canopy height to trunk height is different between patients with depression and healthy individuals (Pintea et al., 2014), however, our studies have not shown significant difference in the ratio of trunk height to the overall height of the tree, because the standard deviation is too high. Larger numbers of patients are needed for future studies to probe into this question.

### Analysis of Canopy of the Tree Roots

There were not statistical differences in root area, width, and height among the three groups, as revealed by one-way ANOVA and LSD-t results. It is suggested that the root of the tree represents the instinct and unconsciousness of the subject (Figure 7). At the same time, some scholars believe that the root of the tree also reflects the cultural heritage of a person (Xie and Ye, 1994), or the traditional culture of the subject’s living environment. The reason for the difference may be due to the fact that most Chinese are influenced by traditional culture and have oppressed instincts, and many people do not draw roots when painting trees. This led to too many missing data points in our statistical analysis. Some previous reports have extensively and profoundly compared both ancient and modern Chinese cultures and found that the vast majority of Chinese people have relatively conservative sexual morality and sexual behaviors (Kaneda et al., 2010).

### Limits of This Study

The data in this study show that, of the 12 quantitative indexes studied, the only statistically significant difference is found in canopy width between the major depressive and subthreshold groups, while the remaining 11 quantitative indexes were not significantly different. This is possibly due to the sample size of the groups. In the future we will add more subjects to test the ratios between the canopy, trunk and roots. In addition, the depressive subjects have had multiple episodes. In future studies, trees drawn by different episodes of major depressive patients should be compared.

In all, our data found that there are differences in the canopy size, width, and height in patients with major depression and those in the control group, indicating that quantitative indexes about the canopy in the tree-drawing test are meaningful in the diagnosis of mental disorders. As far as we know, this is the first report about the objective study of tree drawing, which may play a certain role in the diagnosis of emotional disorders. In addition, tree drawing may provide quantitative aids for clinical diagnosis, and provide a scientific basis for the development of emotional diagnosis norms.

## Ethics Statement

The studies involving human participants were reviewed and approved by the committee of Ethnic Jiangsu University. The patients/participants provided their written informed consent to participate in this study.

## Author Contributions

SG, FW, and WL designed the project, FL, RF, YLiu, GL, YLi, and MG did the experiments. FW, WL, and JH wrote the manuscript.

## Conflict of Interest

The authors declare that the research was conducted in the absence of any commercial or financial relationships that could be construed as a potential conflict of interest.
